# Interaction of Urban Rivers and Green Space Morphology to Mitigate the Urban Heat Island Effect: Case-Based Comparative Analysis

**DOI:** 10.3390/ijerph182111404

**Published:** 2021-10-29

**Authors:** Yunfang Jiang, Jing Huang, Tiemao Shi, Hongxiang Wang

**Affiliations:** 1 School of Urban and Regional Science, East China Normal University, Shanghai 200241, China; jhuang@stu.ecnu.edu.cn; 2 The Center for Modern Chinese City Studies, East China Normal University, Shanghai 200241, China; 3 Research Center for China, Administrative Division, East China Normal University, Shanghai 200241, China; 4 Institute of Spatial Planning and Design, Shenyang Jianzhu University, Shenyang 110168, China; 5 School of Journalism and Communication, Sichuan International Studies University, Chongqing 400031, China; elainewhx@163.com

**Keywords:** urban heat island (UHI), blue–green space, spatial morphology, urban cooling effect (UCI), boosted regression trees (BRT), marginal effect (ME), Shanghai

## Abstract

The spatial morphology of waterfront green spaces helps generate cooling effects to mitigate the urban heat island effect (UHI) in metropolis cities. To explore the contribution and influence of multi-dimensional spatial indices on the mitigation of UHIs, the green space of the riparian buffer along 18 river channels in Shanghai was considered as a case study. The spatial distribution data of the land surface temperature (LST) in the study area were obtained by using remote sensing images. By selecting the related spatial structure morphological factors of the waterfront green space as the quantitative description index, the growth regression tree model (BRT) was adapted to analyze the contribution of various indexes of the waterfront green space on the distribution of the LST and the marginal effect of blue–green synergistic cooling. In addition, mathematical statistical analysis and spatial analysis methods were used to study the influence of the morphological group (MG) types of riparian green spaces with different morphological characteristics on the LST. The results showed that in terms of the spatial structure variables between blue and green spaces, the contribution of river widths larger than 30 m was more notable in decreasing the LST. In the case of a larger river width, the marginal effect of synergistic cooling could be observed in farther regions. The green space that had the highest connectivity degree and was located in the leeward direction of the river exhibited the lowest LST. In terms of the spatial morphology, the fractional cover values of the vegetation (Fv) and area (A) of the green space were the main factors affecting the cooling effect of the green space. For all MG types, a large green patch that had a high green coverage and connectivity degree, as well as was distributed in the leeward direction of the river, corresponded to the lowest LST. The research presented herein can provide methods and development suggestions for optimizing spatial thermal comfort in climate adaptive cities.

## 1. Introduction

With the expansion of urban areas and intensification of the urbanization process, the urban heat island (UHI) effect is becoming a significant urban issue. Natural surfaces, such as vegetation areas and riparian areas, are being replaced by impervious surfaces, resulting in increased long-wave radiation [1,2,3]. Impervious surfaces absorb more solar radiation due to their low reflectivity and high absorptivity, thereby generating a higher land surface temperature (LST) that increases the air temperature, due to the increased long-wave radiation, and induces the urban heat island effect [4,5,6,7]. The high-temperature environment induced by urban heat islands affects human thermal comfort and aggravates air pollution, thereby adversely influencing human health [8,9,10,11]. The urban blue–green space, including waterbodies (rivers, lakes, and reservoirs) and green spaces (parks, gardens, grassland forests, etc.), has a significant ecological function in mitigating UHIs [12,13]. Urban green space can most effectively emit longwave radiation to cool the surface because it has high emissivity and efficiently consumes shortwave radiation through evaporation. At the same time, the high heat capacity attribute and the evaporation latent heat of waterbodies, which can absorb more heat and reduce the ambient temperature, produce the constant cooling effect. Integration of the distribution of blue–green spaces in urban built-up area, therefore, would be essential for the mitigation of the UCI of urban regions [14,15,16].

In the context of the effect of the urban cooling islands of green space (UGCIs), the influence of factors related to the spatial morphology and distribution on the LST reduction, including the area, shape index, green space coverage, building layout, and other environmental elements, has been extensively considered [17,18,19,20]. Green patches with large areas can reduce the surface temperature of green spaces and provide a stable cooling effect [21,22]. Green patches with low landscape shape index (LSI) values are conducive to the cooling effect when the area of green space is smaller than 5.6 ha, but the opposite occurs for larger green spaces [23]. The boundary of green patches has minimal contact with the external environment, thereby facilitating the stable maintenance of the low temperature of the green space [24,25,26]. Green coverage, which is generally evaluated in terms of the normalized difference vegetation index (NDVI) or fractional cover values of the vegetation space (Fv), is negatively correlated with the land surface temperature (LST) in the summer [27,28,29]. Green space with large Fv values has an obvious cooling effect and a complex vegetation structure is helpful to prevent water evaporation [30]. Moreover, the surface albedo, which represents the ability of surface objects to absorb and reflect the surrounding radiation, is negatively correlated with the LST to a certain extent [31]. In addition, the connectivity degree of green spaces considerably influences the cooling effect of such spaces. The DPC index describing connectivity degree can accurately reflect the connectivity level among green patches and inter-connected green spaces provide a higher cooling effect over adjacent areas [32,33].

Recent studies of the impact factors of the waterbody cooling island (WCI) effect have mainly focused on the area, width, and shape of the waterbody and its location in the dominant wind direction [34,35,36]. Based on the ASTER image data of Beijing, the cooling intensity and efficiency of waterbodies were related to its area, geometry, location, and building proportion, as the study examined the cooling effect from 197 waterbodies [25]. The effect of the area of waterbodies on thermal environment is much more significant than that of water depth according to the case study by ENVI-met microclimate simulation [13]. As a linear waterbody, wider rivers have a more notable ability to regulate the thermal environment [37,38]. Moreover, a waterbody exerts a greater cooling effect over the area in the leeward direction [39,40]. Compared with that of the windward area, the cooling intensity of the leeward area was 1.5 °C higher [38]. The temperature difference between the leeward and windward could reach 2 °C in a large area of a waterbody [41].

The blue–green synergistic cooling effect is considerably higher than the single ecological element effect [42,43]. The evapotranspiration of water bodies is stronger under the influence of a green space [44]. Vegetation can affect the water radiation balance, promote air convection, and reduce the off-shore temperature through shading and transpiration [45]. The cooling effect of rivers with a high vegetation coverage is higher than that of rivers with no vegetation [39]. Increasing the vegetation ratio in riparian areas can effectively enhance the cooling effect [46]. The forest buffers provide different efficiencies regarding the reduction of stream air temperature in different periods of summer based on the modelling and analysis of the cooling effect of five rivers in western-Washington state in the summer before and after logging [47]. The maximum temperature difference between the two scenarios with and without rivers around the residential area can reach 1.6 °C based on CFD numerical simulation in Tokyo [48]. A river with a width of 35 m can lead to a decrease of approximately 1–1.5 °C in the ambient temperature and the decrease can be increased if green space is present on both sides of the river [49].The case study of an urban region in Fujian indicated that green spaces adjacent to rivers can exert obvious synergistic cooling effects, which can intensify the cooling effect by approximately 2.7 °C [22]. The green space network pattern in waterfront areas, especially with a high connectivity degree, considerably influences the transportation of cold air in urban rivers [50]. The comprehensive cooling effect of the optimized blue–green space network is greater than that of any single green space element in Shanghai city [42].

In the study of the influencing factors of blue–green spaces on the cooling effect, field measurement, remote sensing technology, and numerical simulation methods are usually used. The field measurement method studies the cooling effect of blue–green spaces by obtaining the temperature data of local specific points [36,39]. However, the measured workload is large and there are many interference factors that affect the measurement results. Numerical simulation is a three-dimensional dynamic simulation based on computational fluid mechanics. At present, the commonly used software platforms are ENVI-met, fluent, WRF, Airpark, etc. Among them, ENVI-met is widely used to simulate the microclimate of green spaces or waterbodies. The maximum simulation grid scale of ENVI-met software is limited to 2 × 2 km, which is more suitable for the micro-scale [13,50]. The technology of RS and GIS can provide continuous LST data at the macro-scale and can also provide overall data for urban meso-scale research. In recent years, it has been widely used in the research field of urban thermal environments [22,35,51].

The focus of research on the cooling island effect is gradually shifting to more complex built-up space environments. The existing studies quantitatively examined the cooling effect correlated to the macro-morphological factors of blue–green spaces or to specific factors at the micro-level. However, the impact of the spatial morphology at the urban meso-level as a controlling index must be more extensively examined for application to urban green space planning [32,52]. The three-dimensional features of the internal structure of green spaces and the features of the surrounding areas influence the thermal environment distribution of green space patches. These spatial environmental indicators must be integrated with the characteristics of the spatial structure and morphological factors to explore the cooling effect. Notably, urban waterfront areas are usually the first preference of urban residents as a public activity space. To maximize the urban cold island effect in the hot season, the spatial pattern of green spaces located on both sides of urban rivers is thus the focus of climatic adaptability planning in urban building areas.

The city of Shanghai is located in a plain river network region, in which many rivers intersect and are connected across the built-up environment. In this study, typical river systems in the study area at the urban-meso-spatial scale in Shanghai were selected. A machine learning algorithm was used to analyze the contribution ratio of each structural and morphological factor to the LST decrease in the waterfront green space and the marginal effect of blue–green synergistic cooling in river corridors. The morphological group (MG) types with notable cooling effects, as classified by the dominant spatial index, were used to identify the morphological characteristics of the waterfront green space considered for microclimate adaptability. Subsequently, the index describing the quantitative spatial aspects could be applied to standardize the green space planning and design practice.

## 2. Study Area and Methodology

### 2.1. Study Area

Shanghai is located at 120°51′–122° E and 30°41′–31°53′ N; this region has a typical subtropical monsoon climate, with hot and humid summers and cold and dry winters. The hot weather in Shanghai occurs in July and August. In 2017, there were 28 days in July and 17 days in August in which the daily maximum temperature in Shanghai exceeded 35 °C [53]. Coupled with intensive land use and high population density in the built-up area, the UHI phenomenon was more significant in the past twenty years. The proportion of UHI area in the jurisdiction area was 9.47% in 2000, which reached 20.49% in 2009 and 34.18% in 2017 [54].

The overall terrain of the city area is a broad and alluvial plain with an average altitude of about 4 m. The rivers within the jurisdiction originate from many lakes and upper reaches on the eastern edge of the Taihu Lake Basin. The river network density of Shanghai is 4.53 km/km^2^ and the waterbody surface ratio of the rivers and lakes is 9.79% [55]. The styles of waterscapes are mainly river corridors, especially the Huangpu River section flowing through the central area from the south to north, which is the most important main river in the study area.

**Figure 1:**
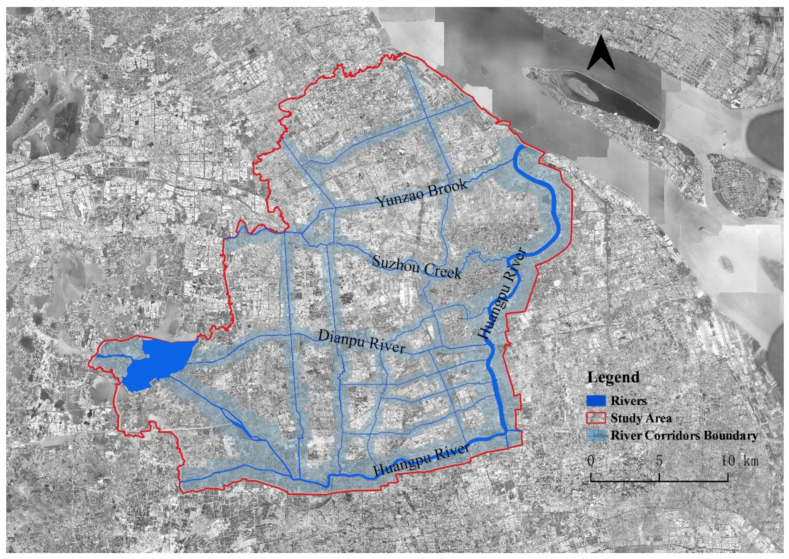
Locations of the backbone river corridors and their river buffer areas in the study area.

A relatively holistic area, which is enclosed by the east–west and north–south segments of the Huangpu River, was selected as the study area. The green patches in the area are ecologically diverse and the waterfront public space is characterized by multiple patterns. Small and medium parks are scattered in the city center, while large green spaces and agricultural land are mostly distributed in the suburbs. Due to the high-density built-up area in the city center, the distribution of the UHIs and the difference in the spatial thermal environments in this area are notable. In the study area, the water system of the backbone river based on the “2017 Shanghai River Channel (Lake) Report” was considered and the river buffer area was identified considering the river width and boundary of the urban blocks and roads (Figure 1).

### 2.2. Data and Methods

#### 2.2.1. Research Framework

**Figure 2:**
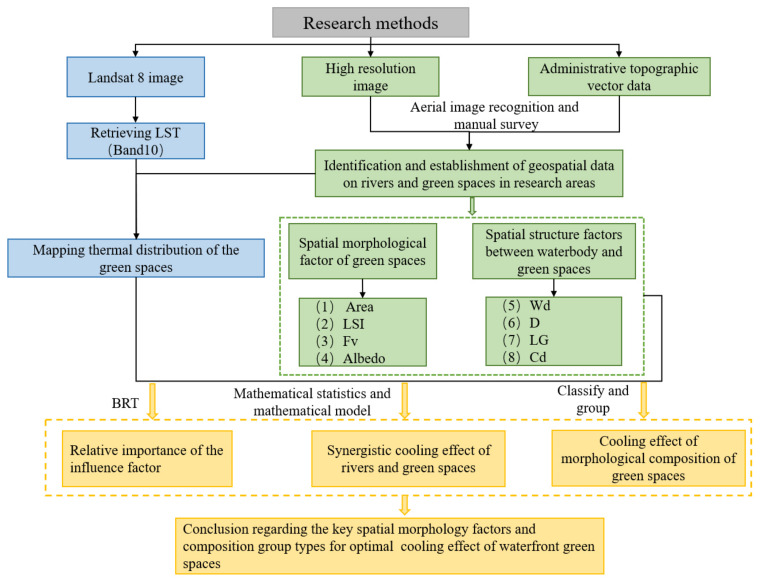
Process flow of the study. BRT: boosted regression trees; LSI: landscape shape index; Fv: fractional cover values of vegetation space; Wd: width of river; D: distance to riverbank; LG: location of greenspace; Cd: connectivity degree.

The methodological framework of this study is shown in Figure 2. The typical process for cooling effect research involves four key steps: (1) The first step involves the retrieval of the land surface temperature in the study area by using a radiative transfer equation (RTE) and extraction of the urban surface temperatures based on band 10 of Landsat 8 remote sensing images. (2) The second step involves the establishment of a geospatial database of the river corridors and waterfront green spaces. High-resolution aerial image data and administrative topographic vector data were combined with manual field surveys to establish a spatial distribution database of both the urban river corridors and waterfront green spaces. Based on the spatial database, the green space morphological index and spatial structure factors between the waterbodies and green space were quantified. (3) The third step involves, the correlation analysis between the LST values and morphological factors of the blue–green space. Using spatial analysis methods based on the BRT model, on mathematical statistics, and on the classification and grouping of spatial data, the influence of the multi-dimensional index factors of the waterfront green space in a river corridor was clarified and the cooling characteristics of the selected spatial morphological factors were analyzed. Based on the cooling effect of these morphological indices, the spatial distribution mechanism of the urban cooling island effect of blue–green spaces was explained.

#### 2.2.2. Land Surface Temperature

To retrieve the land surface temperature, the surface temperature was determined using the ENVI5.3 software. The urban surface temperatures were extracted based on the Landsat 8 TIRS image data obtained by the United States Geological Survey (USGS) at 10:25 on 24 August 2017, under a cloud cover of 0.4%. This process was implemented in two steps. First, the band 10 thermal infrared data of Landsat 8 thermal infrared sensor (TIRS) images were subjected to radiometric calibration and atmospheric correction based on the fast line-of-sight atmospheric analysis of the spectral hypercubes (FLAASH) model in the ENVI software. Second, band 10 of the TIRS image was used to retrieve the land surface temperature (LST). In general, three methods can be used for Landsat-TM data retrieval, namely the radiative transfer equation (RTE), mono-window algorithm (MWA), and single-channel method (SCM). In this study, the RTE based on band 10 was used to retrieve the LST due to its higher accuracy compared to the other methods [56].

The principle of the radiative transfer equation (RTE) is as follows: First, the influence of the atmosphere on the surface thermal radiation is estimated. Next, the atmospheric influence is subtracted from the total amount of thermal radiation observed by the satellite sensors to obtain the surface thermal radiation intensity. This value is transformed to the corresponding surface temperature [51]. The calculation formula is as follows [57]:

(1) $$L\lambda =\left[\varepsilon B\left(Ts\right)+\left(1-\varepsilon \right)L↓\right]\tau +L↑$$

where *L_λ_* is the luminance value of the thermal infrared radiation received by the satellite sensor, *L*↑ is the atmospheric upwelling radiance, *L↓* is the atmospheric downwelling radiance, *ε* is the emissivity of the surface, *Ts* is the true land surface temperature, *B*(*Ts*) is the black body radiation intensity determined using the Plank radiation function, and τ is the atmospheric transmissivity. The three atmospheric profile data points (*τ*, *L↑*, and *L*↓) can be obtained from NASA’s website (http://atmcorr.gsfc.nasa.gov) (accessed on 25 January 2020).

The variables *ε, B(Ts),* and *Ts* can be calculated as follows:

(2) ε=0.004Fv+0.986

(3) $$Fv=\left[\left(NDVI-NDVI_{soil}\right)/\left(NDVI_{veg}-NDVI_{soil}\right)\right]$$

where *Fv* is the fractional cover value of the vegetation space; *NDVI* is the normalized difference vegetation index; *NDVI_soil_* is the *NDVI* value for areas that have completely bare soil or no vegetation cover, considering the empirical value *NDVI_soil_* = 0.05; and *NDVI_veg_* represents the *NDVI* value of the area completely covered by vegetation, considering the empirical value *NDVI_veg_* = 0.70.

(4) $$B\left(T_{S}\right)=\left[L\lambda -L↑-\tau \left(1-\varepsilon \right)L↓\right]/\tau \varepsilon \;$$

(5) $$T_{S}=K_{2}/ln\left(K_{1}/B\left(T_{S}\right)+1\right)$$

where *K*_1_ and *K*_2_ are constants. For the TIRS data of Landsat-8, *K*_1_ = 774.89 (*mWm^−2^sr^−1^ μm^−1^*) and *K*_2_ = 1321.08 (*mWm^−2^sr^−1^ μm^−1^*).

**Figure 3:**
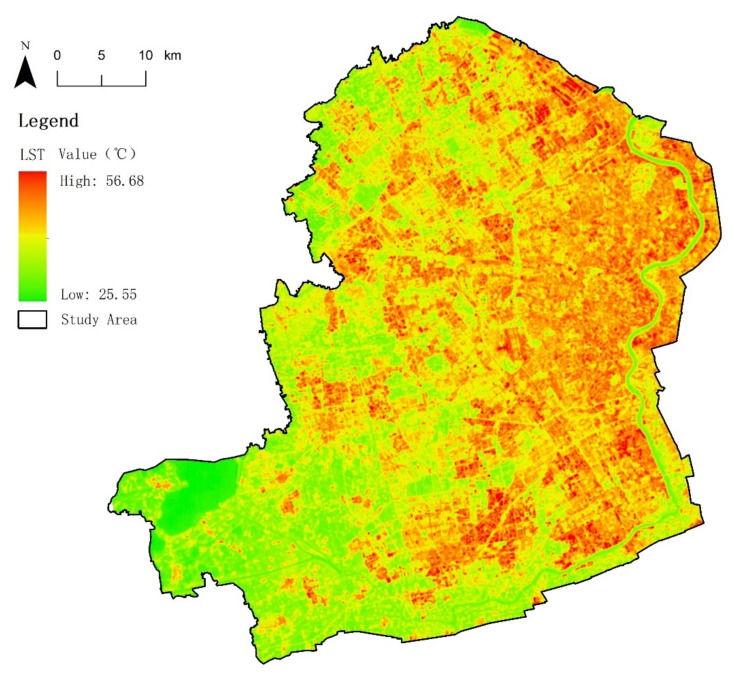
Distribution map of the study area.

The LST distribution map of the study area was obtained (Figure 3) and imported to the ArcGIS10.4 software. The zonal analyst tool of ArcGIS was used to calculate the mean LST value of each green patch.

#### 2.2.3. River Width Classification and Green Space Extraction in the Buffer Zone

Rivers of different widths have different ecological cooling functions. River regulation and land use in waterfront areas are commonly related to the river channel order. A river with an estuary width of less than 30 m is defined as a small and a medium river by the water conservancy department in China [58]. The technical standard for the river classification of plain river networks is 30 m, 30–50 m, 50–70 m, and more than 70 m [59].

In terms of the river morphological factors affecting the cooling effect, studies have shown that the width of the river and the land use along the riverside were the two fundamental factors [25,37]. Previous studies in Shanghai showed that Huangpu River had the lowest temperature zone, which provided a great impact on the thermal environment of Shanghai. The UCI of meso-scale rivers in urban suburbs was relatively obvious. The streams with small widths in the city center had little impact on the surrounding temperature [37]. The relative case study indicated that the LST mean value of Huangpu River (width of 300–770 m) in the 200 m buffer zone was 2.90 °C, 1.69 °C, and 1.01 °C less than that of Suzhou Creek (50–60 m), Yunzao Brook (30–60 m), and Chuanyang River (approximately 70 m), respectively [37]. Moreover, the results of a case study in Beijing city demonstrated that the width of an urban river is a key factor affecting the temperature and humidity effect of the riverside green space [60].

The study of a plain river network in Wuhan city, which has the same climatic conditions as Shanghai, found that the threshold distances of the cooling effect were 550 m, 780 m, 1000 m, 1500 m, and 1700 m in different rivers regions [61]. In this study, using the river hierarchical management data and corresponding research data of different threshold distances, and also considering the notable differences in the river network distribution characteristics and LST value distributions in the study area of Shanghai, four width classifications of urban rivers in the study area were established:

(1) Width-I: the first classification pertains to rivers with a width of less than 30 m and the buffer zone is 500–800 m from the riverbank;
(2) Width-II: the second classification pertains to rivers with a width between 30 and 50 m, and the buffer zone is 800–1500 m from the riverbank;
(3) Width-III: the third classification pertains to rivers with a width between 50 and 80 m, and the buffer zone is 1000–1700 m from the riverbank; and
(4) Width-IV: the fourth classification pertains to rivers with a width greater than 100 m. The width of the river channel in the study area is not in the range of 80–100 m. The buffer zone is 1500–2500 m from the riverbank.

**Figure 4:**
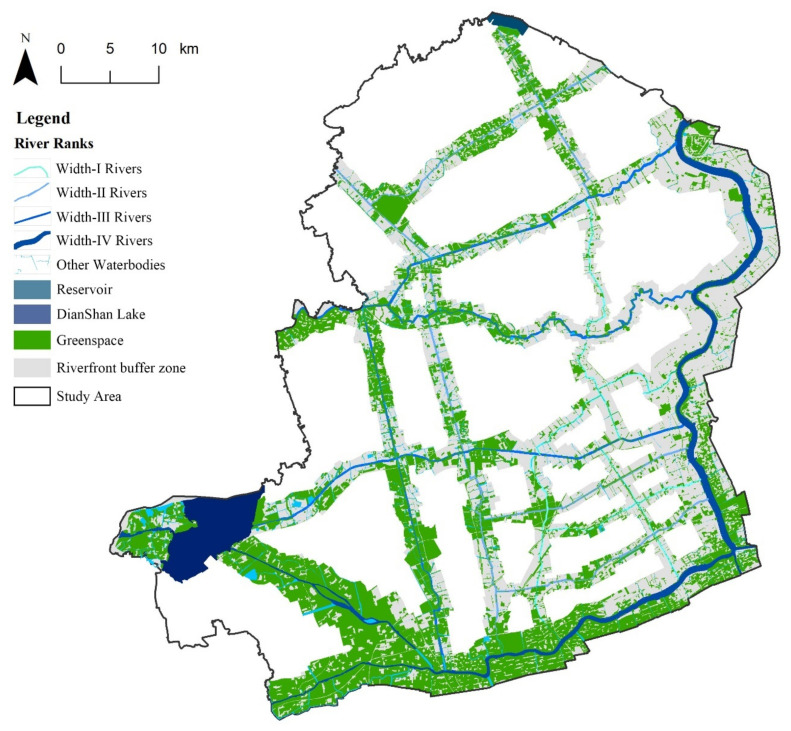
Spatial distribution of blue–green space in the river corridor in the study area.

The green space in the buffer zone was extracted after the river classification. First, based on a high-resolution image of 2017 and vector data of a 1:50,000 road network topographic map of 2015, ground control points (GCPs) were selected to realize the geometric precision correction. Second, the image recognition of the river networks and green space was performed using the method of artificial recognition and field supplementary investigation to determine the spatial distribution of the river networks and each green patch in the study area. Finally, the spatial distribution of the river channels and green spaces in the river corridor, as well as its buffer area, were determined (Figure 4).

#### 2.2.4. Quantification of Multi-Dimensional Spatial Impact Factors of Blue–Green Spaces

The cooling effect of the waterfront green space is related to the spatial morphological factors of the green space as well as to the location of the water body and spatial structure between the blue and green space. In this paper, eight indices were used to describe the spatial characteristics of blue–green landscape patterns (Table 1).

(1) Spatial Morphological Variables

a. Area

The area of the green patches was calculated using the ArcGIS10.4 software platform.

b. Fraction of the vegetation coverage (Fv)

Fv describes the greenness of a green space. The value represents the percentage of the vertical projection area of the vegetation (including leaves, stems, and branches) on the ground to the total area of the statistical area and is a key parameter to describe the vegetation coverage on the ground [62,63]. Fv is the percentage of vegetation reflection in a pixel to the total reflection by decomposition from the interior of a single pixel [64]. The calculation formula is presented as formula (3).

c. Landscape shape index (LSI)

The landscape shape index (LSI) represents the boundary shape of the green space and is determined by calculating the deviation between the shape of a green space patch and a square of the same area [26]. The calculation formula is as follows:

(6) $$LSI=\frac{0.25L}{\sqrt{A}}$$

where *L* is the total perimeter of the green patch and *A* is the area of the green patch.

d. Albedo

The surface albedo is the ratio of the surface reflection flux to the incident solar radiation flux on the surface of a green space [65,66]. Albedo reflects the comprehensive heat radiation impact of vegetation coverage in green space and the three-dimensional shape of adjacent surrounding environments near green space [48]. In this study, the inversion model for Landsat-TM data, as established by Liang, was applied to retrieve the Landsat 8 data to estimate the surface *albedo* [67]. The calculation formula is as follows:

(7) $$Albedo=0.356B_{2}+0.130B_{4}+0.373B_{5}+0.085B_{6}+0.072B_{7}-0.0018$$

where *B*_2_, *B*_4_, *B*_5_, *B*_6_, and *B*_7_ represent the blue, red, near infrared, and both 1 and 2 bands of Landsat 8 data, respectively.

(2) Spatial Structural Variables

e. Location of the green space (LG)

This variable is defined according to the dominant wind direction to determine the position of green space relative to the river, specifically in the windward or leeward direction of the river.

f. Connectivity degree (Cd)

The connectivity degree is an effective index to evaluate the continuity of the landscape spatial structure. The decrease in the probability of connectivity (dPC) was selected to evaluate the connectivity degree of the green space in the whole blue–green ecological network to measure the influence of the connectivity of green space [68]. The Conefor Sensinode 2.6 software was used to calculate the dPC value of each green space.

To calculate the dPC values, first, the probability of connectivity (PC) was calculated and the dPC values were calculated based on the PC. The calculation formula is presented as formulas (8) and (9):

(8) $$PC=\frac{\sum_{i=1}^{n}\sum_{j=1}^{n}a_{i}a_{j}{P_{ij}}^{*}}{A_{L}^{2}}$$

where *n* is the total number of green patches and P_ij_* is the maximum product probability of all possible paths between patches *i* and *j* (including the direct dispersal between the two patches). *a_i_* and *a_j_* are the areas of the habitat at patches *i* and *j,* respectively, and *A_L_* is the total landscape area. The PC values are bounded (ranging from 0 to 1) and defined as a probability of coincidence in a manner similar to the degree of coherence [69].

(9) $$dPC_{k}=\frac{{PC}_{}-{PC}_{\;remove,\;k}}{{PC}_{}}\times 100\%$$

where *PC_remove, k_* is the overall possible connectivity of the remaining patch after the removal of the ‘*k*’ green patch. *dPC* measures the importance of the patches in maintaining the landscape connectivity through changes in PC [70].

g. River width (Wd)

This value is the mean course width of each green patch adjacent to the river segment. The value was calculated using the ArcGIS10.4 software. Subsequently, MATLAB 2019 was used to determine the mean width by considering the area and perimeter of each river segment.

h. Distance of the waterfront green space from the riverbank (D)

This value is the smallest geometric distance from the green center to the riverbank. The geometric center point of the green patch was extracted using ArcGIS10.4 [71]. Subsequently, the near analysis tool in ArcGIS10.4 was used to obtain the distance from the river.

#### 2.2.5. Analysis of the Influence of Spatial Morphological Structure Factors

(1) Boosted Regression Tree (BRT) model

The boosted regression tree (BRT) model is a machine learning method that combines the advantages of regression and growth models [72]. In terms of output results, the relative influence (or contribution) of each variable is scaled so that the sum is added to 100. The larger the value, the greater the correlation with the dependent variable. The BRT model can also simulate the marginal effect of independent variables and reflect the influence threshold of independent variables on dependent variables in different intervals [73,74]. The BRT model has been widely used to study urban expansion and its influencing factors, and to identify the cold air path and most important predictive variables of cold air path occurrence [73,75]. In recent years, the BRT model has also been applied in the context of the urban heat island effect to study the correlation between the urban heat island effect and the influencing factors pertaining to both urban two-dimensional and three-dimensional indices, such as the normalized difference vegetation index (NDVI), normalized difference built-up index (NDBI), sky view factors (SVF) and building height [76].

In this study, the BRT model was used to analyze the contribution of the eight structural and morphological variables of waterfront green spaces with the different width classifications of urban rivers to the LST values of green patches. The causal variable was the LST of green patches, and the independent variables were the descriptive spatial variables. The decision tree complexity, learning, rate, and split ratio were 5, 0.01, and 0.5, respectively, and Gaussian data were adopted. This model extracted 50% of the data points for analysis each time, with 50% of the data used for training, and 10-fold cross-validation was performed to estimate the number of optimal trees [50,75,76]. The contribution ratios of each factor by BRT regression reflected the importance of independent variables to LST distribution; The marginal effect (ME) changes presented the UCI threshold values and correlation characteristics of each factor.

(2) Criteria for threshold of marginal effect analysis

The marginal effect (ME) curve between D factor and LST values were used to analyse the affecting rang of synergistic cooling effect of blue–green space. For exploring the important correlation of distance intervel to cooling effect, it is necessary to determine the numerical significance of the following basic criteria and important inflection points of the ME:

a. The inclination of ME curve: The inclination of the ME curve represented the severity of the changes in marginal utility. When the curve took on an ascending trend with great inclination degree, the marginal utility increase was very large; When the curve took on an ascending trend with gradual inclination degree, the marginal utility increase was weak; When the curve inclination was a declining trend, the marginal effect of cooling effect was decrease.
b. The optimal distance of marginal utility: The first inflection point where the inclination degree of the ME curve changed from great ascending trend to gradual ascending trend. It indicated that the marginal utility of synergistic cooling effect of blue–green space was in the optimal growth state, and the corresponding D value is the optimal distance with the most economic utility of blue–green space to reach the good cooling effect.
c. The maximum distance of marginal utility: This inflection point was the peak values of the marginal effect curve from an ascending trend to a declining trend. It represented the maximum value of the synergistic marginal utility of blue–green space. When the curve was in the gradual ascending range, the marginal utility of waterbody cooling effect declined with the increase of distance, and the ME of green space continues to produce cooling effect. The holistic synergistic cooling effect of riverfront blue–green space increased slowly and reached the maximum cooling effect at the inflection point.
d. The threshold distance of cooling effect: The ME curve showed a declining trend, and after it declined to the lowest value, the curve appeared irregular alteration. This inflection of the lowest value represented the longest distance of the blue–green synergistic cooling effect. The declining interval of ME curve presented the ME attenuation of cooling effect from both blue space and green space. The position of the lowest attenuation value of the curve was no longer affected by the distance from the synergistic ME of blue–green spaces, namely, the threshold distance of the blue–green synergistic cooling effect was identified.

(3) Classification of spatial morphological group types (MG types) and correlation analysis for the LST

The impact of the spatial factors of a waterfront green space on the LST is a comprehensive effect. The different intervals of a single spatial independent variable usually lead to significant spatial differentiations of the causal variable LST. A single spatial variable can be graded by the corresponding LST influence change interval and multiple spatial independent variables can be composed as well as constituted as by certain MG types, which are grouped by the basic grade elements of single factors in every possible combination to describe the classification of green patches with certain spatial three-dimensional characteristics. Different three-dimensional characteristics of green patches produce corresponding differences in the LST distribution. This kind of partitioning of the MG types to describe the morphology composition of green space can clearly reflect the cooling effect of green space types in terms of the spatial and morphological characteristics. This key innovation distinguishes our study from the existing research.

The four key factors affecting the LST, namely the area, FV, Cd, and LG, were used to construct the morphological group (MG) types. Considering that the threshold cooling distances of different width grades of rivers are different and to reduce the interference of specific surrounding environmental factors in the study area far from the rivers, the distribution data of the waterfront green space at a distance of 200 m from the riverbank were selected to identify the great cooling effect correlation between the MG types and LST values.

**Figure 5:**
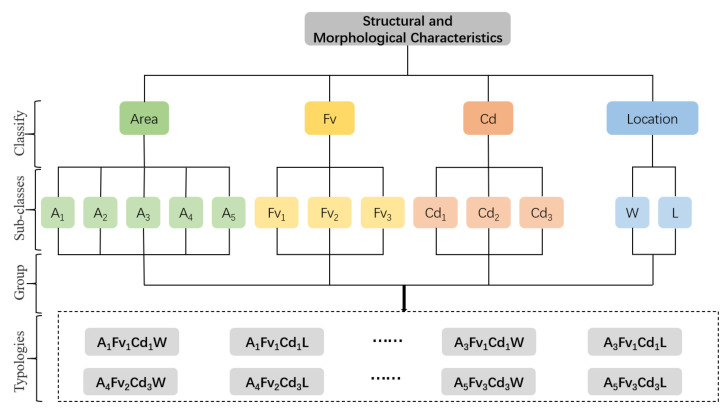
Logical division and classification of green space morphological types.

The classification method of MG types and the analysis process of the correlation characteristics between MG types and LST were as follows:

- The grade level of a single spatial variable was classified (Table 2). Each spatial variable was assigned different grade levels. The value interval was identified based on the effect of the variables on the spatial differentiation interval of the LST values.
- The MG types were grouped. A subcategory of each factor was randomly combined to form MG types with different structural and morphological characteristics. The specific combinational logic and type delimitations are illustrated in Figure 5.

c. The correlation characteristics between the MG types with great cooling effects and LST values were identified. The temperature standard of highly suitable green spaces was considered to reflect the residents’ general body temperature that can meet the survival needs of residents. The standard of high-temperature heat waves determined by the Chinese government involves a maximum daily temperature of 35 °C as the limit for green space cooling optimization. The results show that in August, the difference between the LST and air temperature is approximately 1.8 °C [77]. In this study, the data group with an LST lower than 36.8 °C was selected. According to the increasing sequence of the LST, the correlation characteristics between different Mg types and LST were analyzed by observing the classification of the spatial composition of the corresponding MG types.

## 3. Results

### 3.1. Width-I Rivers (20–30 m)

#### 3.1.1. Contribution of Each Spatial Variable to the LST

**Figure 6:**
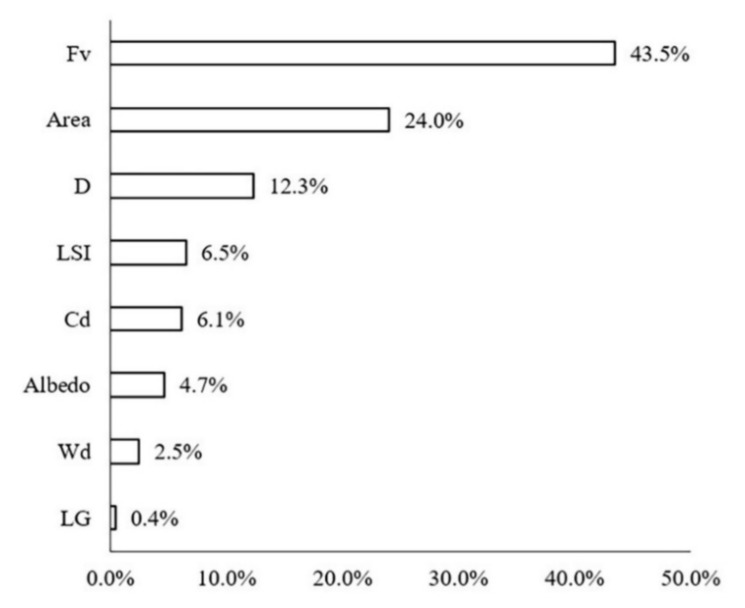
Contribution ratios of each spatial variable to the LST in Width-I river corridors.

The contribution ratios of various impact factors of the green space to the LST were calculated in the buffer zone of Width-I rivers by using the BRT model (Figure 6). Fv (43.5%), area (24.0%), D (12.30%), LSI (6.5%), and Cd (6.1%) were noted to be the most important impact variables. The Fv, area, and LSI were used to describe the morphology of green space. For Width-I rivers, the composition and shape of the green space were the dominant factors. Specifically, the total contribution ratio of the Fv and area was as high as 67.50%, which indicated that large-scale vegetation coverage had a significant impact. The contribution of D to LST was notable, as well, with a contribution ratio of 12.3%. The contribution ratios of the other factors were extremely low and LG exerted the smallest influence (0.4%).

#### 3.1.2. Relationship between D (Distance of the Waterfront Green Space from the Riverbank) and LST Values

**Figure 7:**
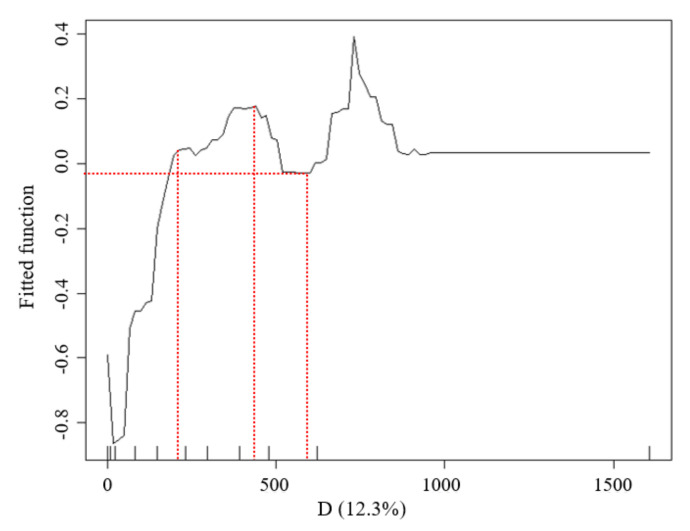
Relationship between the D factors and LST of the waterfront green space in the buffer zone of Width-I river corridors.

The LST data distribution of the waterfront green space in Width-I rivers was considerably affected by the D values (Figure 7). The marginal effect (ME) curve obtained by the BRT model showed that (1) when D was less than 250 m, the inclination of the marginal effect (ME) curve changed significantly, while the inflection point at 250 m represented the distance with the most economically marginal effect. In other words, the UGCI located within 250 m strengthened the WCI effect, resulting in the best combination of the synergistic cooling effect. The curve inclination was large, which illustrated that the interaction between the two cold islands was significant. (2) When the D ranged from 250 to 400 m with increasing distance, the cooling effect intensity of the waterbody decreased and the synergistic cooling effect of the blue–green space gradually increased, with the total effect maximized at 400 m. (3) When D was between 400 and 600 m, the inclination of the ME curve decreased, indicating that the influence of the WCI had reduced. The ME was the lowest at 600 m, which was identified as the threshold distance of blue–green synergistic cooling in the Width-I river zone.

#### 3.1.3. Morphological Group (MG) Types and LST Values

**Figure 8:**
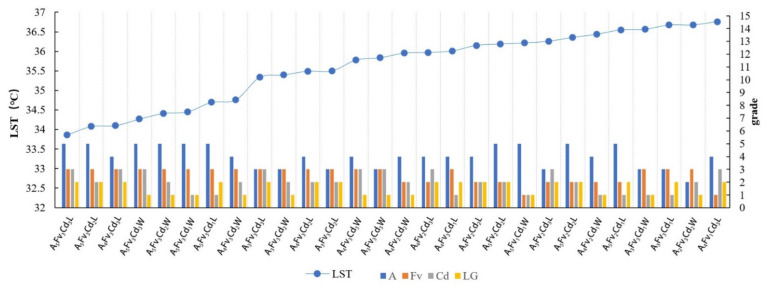
Sorted MG types with LST values lower than 36.8 °C in the Width-I river buffer zone and the linear correlation between the MG types and their LST values.

The MG types with LST values lower than 36.8 °C were observed and sorted according to the increasing sequence of LST values. Most of the MG types with low LST values corresponded to high levels of Fv and A (Figure 8). Width-I rivers corresponded to the least number of ME types with LSTs below 36.8 °C, which indicated a certain influence of the river width. For values lower than 35.5 °C, the variation degree was notable and the LST changed considerably with the MG types. The MG types of Fv_3_ and A_5_ had the lowest LST. For these MG types, the influence of the Fv factor was greater than that of the area. For ME types with the same Fv and area classifications, a higher Cd grade corresponded to a lower relative LST temperature. The cooling effect of the MG types in leeward locations was generally higher than that of the MG types in windward locations.

**Figure 9:**
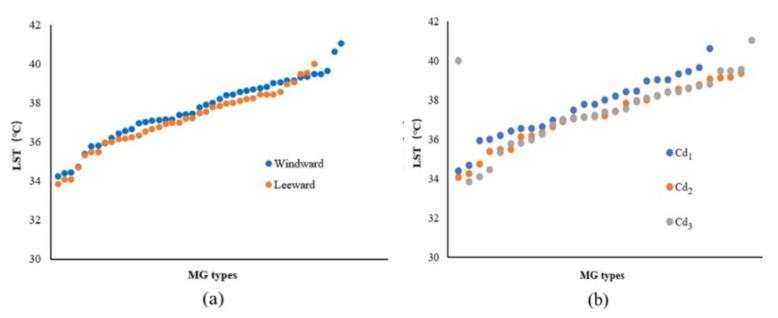
(**a**) Scatter plot of LST values corresponding to the MG types of green space in different locations (leeward or windward) in the Width-I river zone according to the increasing LST. (**b**) LST values corresponding to the MG types of green space in different Cd grades in the Width-I river zone according to the increasing LST.

According to the 81 groups of MG-type data in the 200 m range from the riverbank, with a strong cooling influence zone, the change characteristics of the LST values for the ME types in different locations (leeward or windward) were analyzed according to the increasing LST. In the leeward zone, the MG types usually exhibited low LST values and evident differences were observed between the L and W locations (Figure 9a). The mean temperatures for the leeward and windward MG types were 37.11 °C and 37.57 °C, respectively. The ascending order of the corresponding LST values of MG types with Cd levels indicated that the green space with higher-grade Cd usually exhibited lower LST values compared to those of lower-grade Cd (Figure 9b). The mean LSTs of Cd_1_, Cd_2_, and Cd_3_ were approximately 37.59 °C, 37.28 °C, and 37.36 °C, respectively.

### 3.2. Width-II Rivers (30–50 m)

#### 3.2.1. Contribution of Each Spatial Variable to the LST

**Figure 10:**
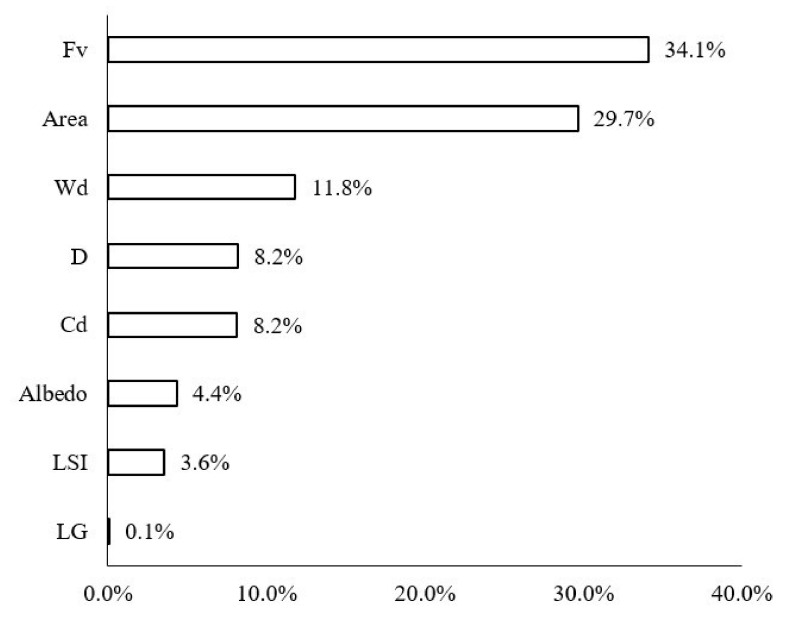
Contribution ratios of each factor to the LST of the waterfront green space in Width-II river corridors.

The contribution ratios of the green space factors in the buffer zone of the Width-II rivers to the LST changed more notably than those of the Width-I rivers (Figure 10). Although the factor that most notably influenced the LST values was Fv (34.1%), followed by A (29.7%), the importance of the river width increased and it emerged as the third most-notable influencing factor, with a contribution ratio of 11.8%. Furthermore, D and Cd notably influenced the LST and their contribution ratios reached 8.2%.

#### 3.2.2. D Factors and LST Values

**Figure 11:**
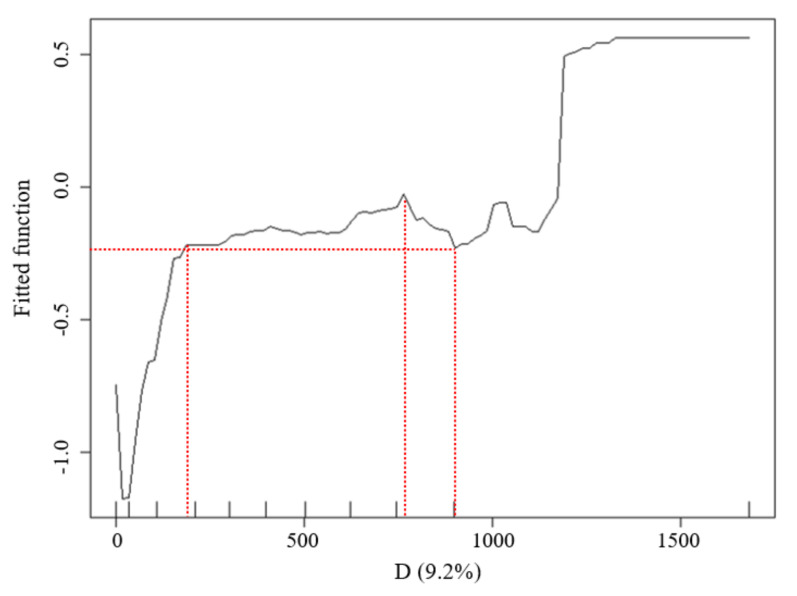
Relationship between the D factors and LST of the waterfront green space in the buffer zone of Width-II river corridors.

The LST data distribution of the waterfront green space in the Width-II river zone was highly influenced by the D values (Figure 11). With the increasing river width, the cooling effect of the green space was observed over larger distances. When D was less than 205 m, the inclination of the marginal effect (ME) curve was large and a considerable synergistic cooling island effect was observed between the waterbody and the green space. The inflection point at 205 m represented the D with most economically marginal effect; when D was between 205 and 765 m, the ME of the blue–green synergistic cooling island effect increased slowly and reached a maximum value at 765 m. When D was between 765 and 900 m, the inclination tendency of the ME curve decreased. Additionally, the value was minimized at 900 m, which was the threshold distance of blue–green synergistic cooling in the width-II river zone. When D was greater than 900 m, the WCI from the rivers did not affect the surrounding environment and the UGCI from the green space was dominant.

#### 3.2.3. MG Types and LST Values

**Figure 12:**
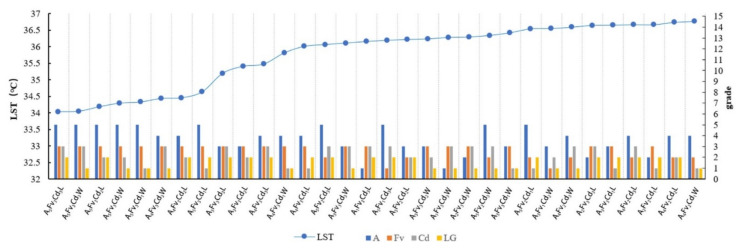
Sorted MG types with LST values lower than 36.8 °C in the Width-II river buffer zone and the linear correlation between the MG types and their LST values.

For Width-II rivers, the numbers of ME types corresponding to LST below 36.8 °C increased and the change in the LST value of each MG type was more gradual than that of Width-I (Figure 12). The Fv and area factors remained the dominant factors. The MG types with high-grade levels of Fv and area factors exhibited low LST values. The LST variation gradient of the MG types was relatively larger under values lower than 35.5 °C, although the variation range of the LST was narrower than that for the Width-I rivers. The effect of the Cd factor on the LST was enhanced and the MG type of green space with a low LST exhibited a high connectivity.

**Figure 13:**
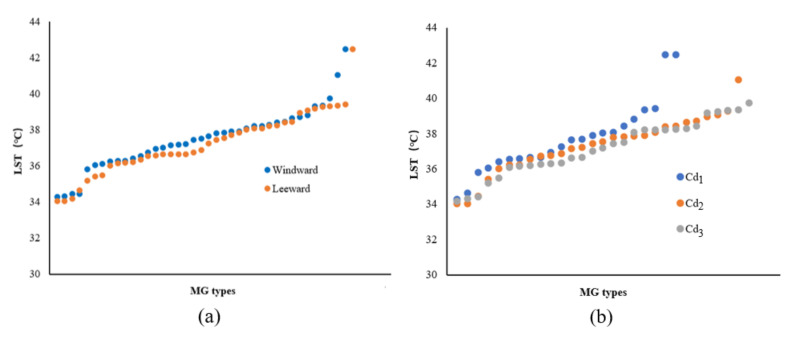
(**a**) Scatter plot of LST values corresponding to the MG types of green space in different locations (leeward or windward) in the Width-II river zone according to the increasing LST. (**b**) LST values corresponding to the MG types of the green space with different Cd grades in the Width-II river zone according to the increasing LST.

Based on the LST data of the MG types located 200 m from the riverbank with a strong cooling influence zone, the variation in the LST value of the ME types in different locations (leeward or windward) was analyzed. The MG types located in the L-direction exhibited low LST values and the LST differences for the MG types in both the leeward and windward directions were smaller than those of the LST values for the corresponding Width-I cases (Figure 13a). The mean LSTs of the MG types in the leeward and windward directions were 37.25 °C and 37.45 °C, respectively. Notable differences were observed in the LST values among the ME types with three Cd levels. The MG types of green spaces with lower-grade Cd exhibited lower LST values (Figure 13b). The mean LSTs of Cd_1_, Cd_2_, and Cd_3_ were 37.67 °C, 37.37 °C, and 37.08 °C, respectively.

### 3.3. Width-III Rivers (30–50 m)

#### 3.3.1. Contribution of Each Spatial Variable to the LST

**Figure 14:**
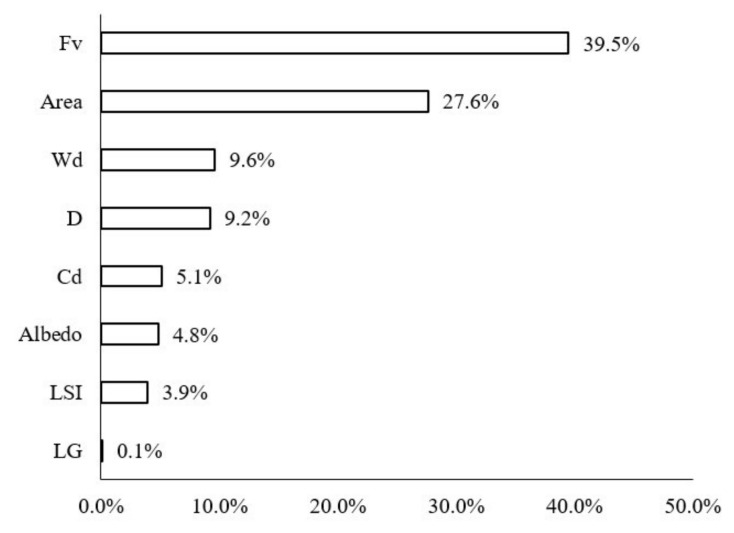
Contribution ratios of each factor to the LST of the waterfront green space of Width-III river corridors.

The contribution of each impact factor to the LST in the waterfront buffer zone of the Width-III rivers was consistent with that of the Width-II rivers (Figure 14). FV, area, Wd, D, and Cd remained the dominant factors. The influence of three of these factors, i.e., FV, area, and Wd, was significant, with contribution rates of 39.5%, 27.6%, and 9.6%, respectively. With the increase in the river width, the trend was different from that of the Width-II rivers: the D variable was the fourth most-important factor influencing the LST, with its contribution ratio slightly increased to 9.2%. The importance of Cd decreased to 5.1% and the effects of the albedo, LSI, and LG were small but relatively stable.

#### 3.3.2. D Factors and LST Values

**Figure 15:**
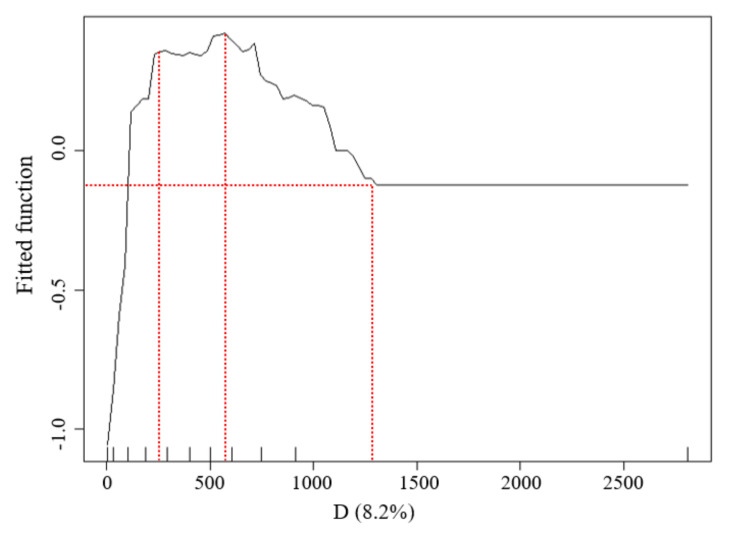
Relationship between the D factors and LST of the waterfront green space in the buffer zone of Width-III river corridors.

The LST data distribution of the waterfront green space in the Width-III river zone was more highly influenced by the distance from the riverbank than that of the Width-II rivers (Figure 15). According to the change in the curve inclination, when D was less than 260 m, the inclination of the marginal effect (ME) curve was large. Namely, with increasing distance, the cooling effect intensity of the waterbody and green space increased significantly and a great synergistic cooling island effect was observed between the waterbody and the green space. The D with the most economically marginal effect of blue–green synergistic cooling was 260 m; when D was between 260 and 630 m, the ME of the cooling effect of the blue–green space increased slowly and reached a maximum value at 630 m. When D was between 630 and 1200 m, the inclination tendency of the ME curve decreased. The lowest value at 1200 m was the threshold distance of blue–green synergistic cooling in the Width-III river zone.

#### 3.3.3. MG Types and LST Values

**Figure 16:**
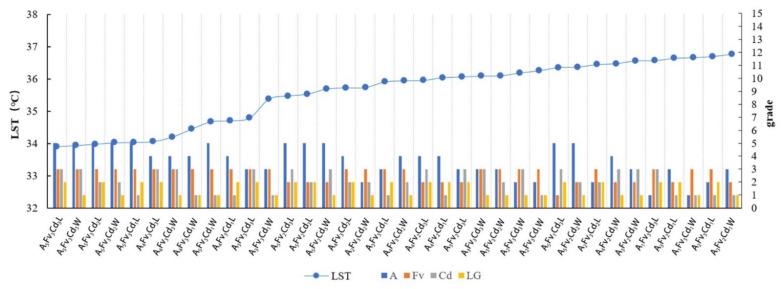
Sorted MG types with LST values lower than 36.8 °C in the Width-III river buffer zone and the linear correlation between the MG types and their LST values.

For Width-III rivers, the numbers of ME types corresponding to LST values below 36.8 °C increased and the gradient changes of the LST values corresponding to each MG type were gentler than those of Width-II rivers (Figure 16). With the aggravation of WCI from the rivers, ME types with lower area-grades existed in the low-value range of the LST. The Fv factor was a notable influencing factor, with high Fv-grade MG-types exhibiting considerably low LST values. The numbers of ME types with LST values below 35.5 °C increased significantly and the proportion of ME types in the low LST range (LST below 35.5 °C) accounted for 30.56% of the total value range (LST below 36.8 °C). In particular, more MG types of green space existed with extremely low LST values. However, the LST change gradient was small and the LST did not change considerably between the different MG types. The effect of the Cd factor on the LST decreased.

**Figure 17:**
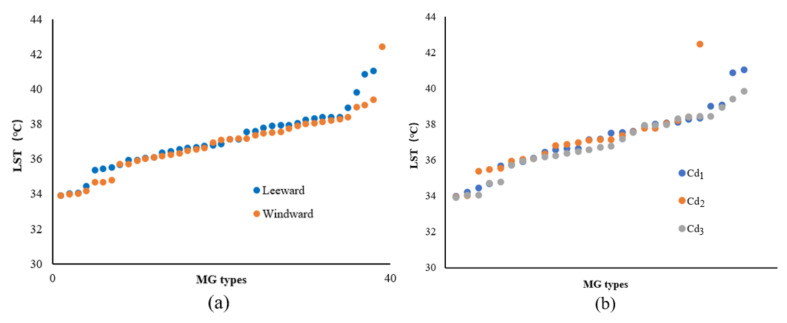
(**a**) Scatter plot of the LST values corresponding to the MG types of green space in different locations (leeward or windward) in the Width-III river zone according to the increasing LST. (**b**) LST values corresponding to the MG types of green space with different Cd grades in the Width-III river zone according to the increasing LST.

According to the LST data of the MG types impacted by a strong cooling distance of 200 m from the riverbank, the influence of the WCI in the Width-III river zone was strengthened and the LST values of most MG types were lower than those for the previous river-width level. The value of the MG types in the leeward area was lower than that in the windward area. The differences in the LST values between the windward and leeward locations were extremely small on both sides of the Width-III rivers (Figure 17a). The mean LST values of the MG types in the leeward and windward locations were 36.91 °C and 37.01 °C, respectively. A negative correlation was observed between the Cd and LST, although the LST differences among the ME types of the three Cd levels were not obvious (Figure 17b). The mean LSTs of Cd_1_, Cd_2_, and Cd_3_ were 37.21 °C, 36.93 °C, and 36.73 °C, respectively.

### 3.4. Width-IV Rivers (Width of More than 100 m)

#### 3.4.1. Contribution of Each Spatial Variable to the LST

**Figure 18:**
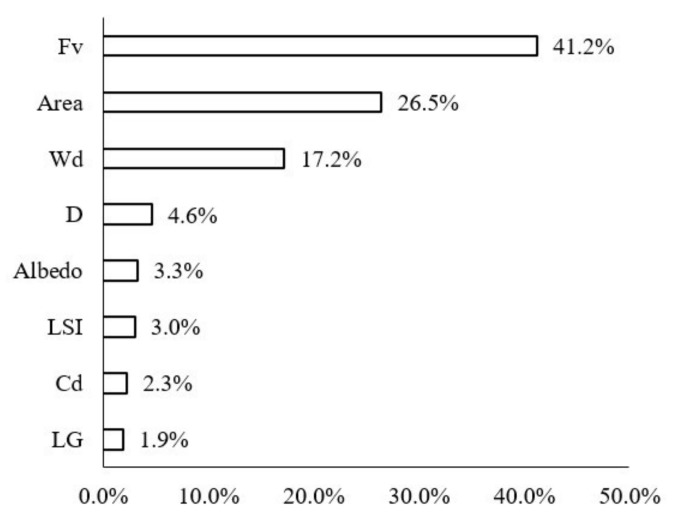
Contribution ratios of each factor to the LST of the waterfront green space of Width-IV river zones.

The contribution of each impact factor to the LST in the waterfront buffer zone of the Width-IV rivers was consistent with that of the Width-II rivers (Figure 18). Fv, area, and Wd were the dominant factors influencing the LST. The contribution ratios of Fv, area, and WD were 41.2%, 26.2%, and 17.2%, respectively. The sum ratio of the three main spatial factors for cooling was approximately 85%. In particular, the proportion of the effect of Wd increased to 17.2%. The importance of D factors on the LST values of the waterfront green space was reduced by 4.6% due to the large range of cooling effects for these rivers.

#### 3.4.2. D Factors and LST Values

**Figure 19:**
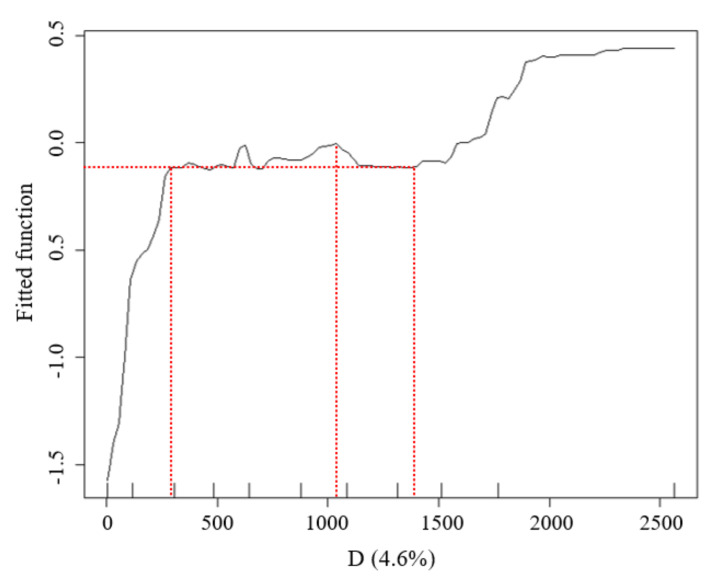
Relationship between the D factors and LST of the waterfront green space in the buffer zone of Width-IV river corridors.

In the Width-IV river zone, the effect of the Wd factor was the highest (Figure 19). According to the inclination changes in the ME curve, when D was less than 250 m, a notable synergistic cooling island effect was observed between the waterbody and green space. When D was between 250 and 1030 m, the ME of the blue–green synergistic cooling island effect increased gradually and reached a maximum value at 1030 m. When D was between 1030 and 1395 m, the ME between D and LST decreased, reaching the lowest value at 1395 m. Namely, the threshold distance of the blue–green synergistic cooling in the Width-IV river zone was 1395 m. When D was more than 1395 m, the LST values increased mainly through the cooling effect of the green space.

#### 3.4.3. MG Types and LST Values

**Figure 20:**
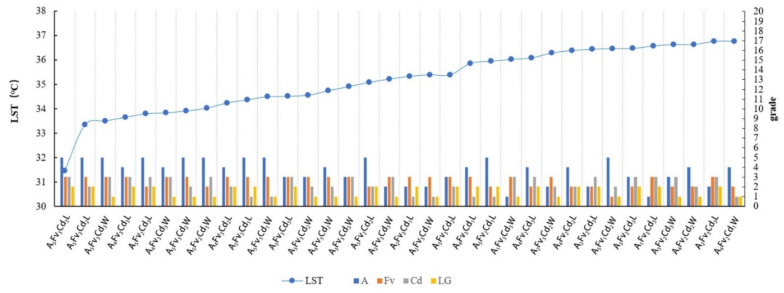
Sorted MG types with LST values lower than the 36.8 °C Width-IV river buffer zone and the linear correlation between the MG types and their LST values.

The amplification of the LST values was the gentlest in the case of the Width-IV rivers and the data of the MG types corresponding to LST values lower than 36.8 °C were selected (Figure 20). The Width-IV zone involved a certain amount of cultivated land that included the waterfront green space, which weakened the cooling effect related to the area factor. As the river width increased, the WCI intensified, resulting in the lower LST of the MG types in the waterfront green space. The Fv factor was significant and the highest Fv grade corresponded to the main body of the MG types with LST values lower than 36.8 °C. The numbers of MG types with values below 35.5 °C were particularly large and the proportion of MG types in the low LST value section (with the LST smaller than 35.5 °C) accounted for 50% of the total LST value section (with the LST smaller than 36.8 °C). Specifically, half of the MG types of the green space exhibited an extremely low LST. The LST variation gradient of the MG types was relatively small. The different Cd grades did not significantly influence the LST temperature.

**Figure 21:**
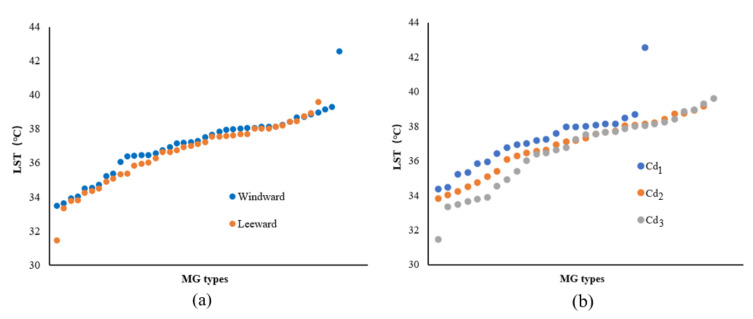
(**a**) Scatter plot of LST values corresponding to the MG types of green space in different locations (leeward or windward) in the Width-IV river zone according to the increasing LST. (**b**) LST values corresponding to the MG types of the green space with different Cd grades in the Width-IV river zone according to the increasing LST.

According to the LST data of the MG types in the 200 m range from the riverbank, with a strong cooling influence zone, the influence of the Width-IV rivers was significant in general and the LST of most MG types was lower than that of the previous three grades of river widths. The LST values of the MG types in the L-location were lower than those in the W location and the difference between them was notable (Figure 21a). The mean LSTs of the MG types in the leeward and windward directions were 36.67 °C and 37.01 °C, respectively. Compared to the LST corresponding to the MG types of the three Cd grades, high Cd values and a low LST distribution correlation were observed among the ME types, and the LST differences for Cd_1_, Cd_2_, and Cd_3_ were significant (Figure 21b). The mean LSTs for Cd_1_, Cd_2_, and Cd_3_ were 37.37 °C, 36.85 °C, and 36.41 °C, respectively.

## 4. Discussion

### 4.1. Difference in the Cooling Effect of the River Width Scale

**Figure 22:**
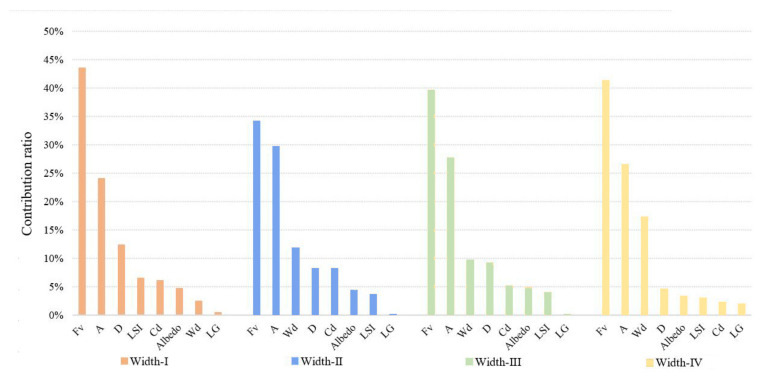
Contribution ratios of each spatial variable to the LST in rivers with different width grades.

The contribution ratios of the Wd factor to the LST were 2.5% (width-I), 11.8% (width-II), 9.6% (width-III), and 17.2% (width-III) according to the BRT model analysis. Based on the importance order of the impact between Wd and LST factors, the importance of Wd was not significant for river widths below 30 m. For rivers with a width of more than 30 m, the contribution of Wd was significant (Figure 22). According to the curve analysis of the relationship between the impact distance of Wd at different grades and the marginal effect of the LST, the distance with a maximum ME of the synergistic cooling effect of the blue–green space in the Width-I river zone was 400 m and its threshold distance was 600 m. The distances with maximum ME of the blue–green space in the Width-II, Width-III, and Width-III rivers were 765 m, 630 m, and 900 m, respectively, and the threshold distances were 900 m, 1200 m, and 1395 m, respectively.

The distribution status of the green space in the waterfront zone of the Width-II and Width-III rivers slightly affected the contribution ratios in this study. The maximum impact range of the WCI was larger for Width-III rivers than that for the Width-II rivers. Moreover, the curve inclination of the Width-III rivers was larger than that of the Width-II rivers, which indicated that the intensity of the blue–green synergistic cooling effect was considerably higher than that of the second-level rivers. Therefore, a larger river width corresponded to a greater cooling effect. In general, a river width of more than 30 m was a notable cooling source for the city and the river width exhibited a gradually increasing influence on the contribution ratio of the cooling effect. In terms of the numerical relationship of the cooling intensity, the UCI for a large river width fluctuated slightly and was relatively stable.

### 4.2. The Importance of Greenspace Morphological Factors in Waterfront Areas

Authors should discuss the results and how they can be interpreted both from the perspective of previous studies and of the working hypotheses. The findings and their implications should be discussed in the broadest context possible. Future research directions may also be highlighted.

Through the BRT model analysis and comparative study, and considering the background of the river corridors with different grade widths, it was observed that the influence of the green space morphology on the LST in waterfront areas was significant. The research results showed that the effect of the morphological factors on the LST distribution was greater than that of the rivers. This feature could be clearly identified considering two aspects:

- In the index system of the LST-related morphological factors of waterfront green spaces, Fv and area considerably influenced the LST. The total contribution ratio of the two factors was more than 60% and Fv was the primary factor affecting the LST, as observed in the previous studies [18].
- In the river width classification studies, the D factor exhibited a negative correlation with the LST. In particular, the regression relationship between D and LST, and the ME was below zero for the Width-II and Width-IV rivers. This finding reflected the fact that the water cold island effect decreased with increasing D, but the synergy of the blue–green space increased the intensity of the marginal effect. When a constant negative correlation existed between the two aspects, the UGCI strengthened the UWCI, and the cooling values exceeded the attenuation values when D was large.

**Figure 23:**
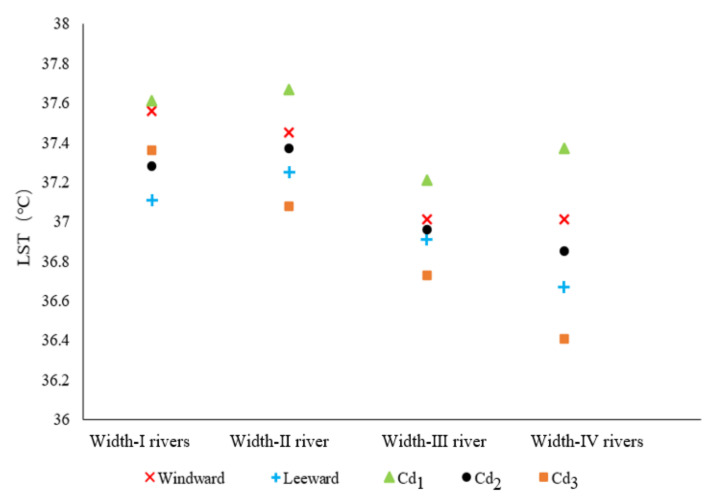
Correlation analysis between LST and LG or Cd factors of different grades of rivers.

Furthermore, the morphological factors between the waterbodies and green spaces or among green space systems influenced the cooling effect. The LG factors and Cd relationship among the green space systems were analyzed (Figure 23). With the increase in the river-width grade, the LST values of the waterfront green space, according to the classification of the LG and Cd types, decreased. The LST values of the green space in the L-location were lower than those in the W-location and the LST values of green space with high Cd-types corresponded to the lowest values. Moreover, the comparative analysis of the Cd contribution ratio indicated that the impact of the Cd factors in the Width-I and Width-II river zones was significant. As the river width increased, the Cd effect attenuated, and the relative effect of Cd was quite low in the Width-IV river zone. Therefore, for small and medium rivers, the improvement of the green space connectivity degree is necessary to maximize the cooling island effect in the holistic waterfront area layout.

Regardless of the width grade of the river, the MG types with the lowest LST were concluded to be A_5_Fv_3_Cd_3_L (Figure 8, Figure 12, Figure 16, and Figure 20), with LST values below 34 °C. In addition, the MG types of A_5_Fv_3_Cd_3_W, A_5_Fv_3_Cd_2_L, and A_4_Fv_3_Cd_3_L exhibited a low LST. Therefore, MG types with the characteristics of large areas, high vegetation coverage, and high connectivity degrees were noted to have the highest cooling effect. The blue–green synergistic cooling effect was key to solving the thermal environment problems in plain river network cities during hot summers. A waterfront greenbelt with a certain width and high vegetation coverage can enhance the cooling synergistic effect, particularly in the D range with the maximum ME. Additionally, the moderate distribution of a large green space in the buffer zone and the formation of a continuous green space network can help induce a more significant blue–green synergistic cooling effect.

### 4.3. Differences Compared to the Existing Studies

In previous studies on the impact of distribution pattern characteristics of waterbodies and green spaces based on remote sensing images in thermal environments, raster data and the grid analysis method were mostly used to quantify the composition of land cover types (including water body and vegetation coverage) and other elements in the unit grid space. Then, conduct correlation analysis was conducted with their corresponding land surface temperature so as to obtain the influencing characteristics of the main cooling efficiencies of waterbodies and different types of green spaces [22,25,32]. For urban spatial planning and design, specific waterfront green spaces represent the spatial units. Their quantitative controlling of spatial morphological indices was aimed at providing the direct policy-making method for forming the best waterfront cooling network from the aspects of holistic spatial composition and morphology, as well as from the aspect of their spatial structure characteristics in the riverfront region. As there is a lack of real descriptions on the spatial attributes of actual features, it is obvious that unit raster data cannot provide direct and effective suggestions for spatial development. Our research formed a quantitative description of waterfront green spaces and analyzed the cooling correlation of spatial factors.

The BRT model has strong adaptability to datasets and can handle both continuous and categorical data, and can reflect the comprehensive interaction of variables. The cooling effect of the influencing factors of blue–green space depends on the morphological composition and spatial pattern elements, which has been verified by a large number of preliminary studies [49,50]. The correlation methods primarily analyzed the unilateral effect of indicators in an independent manner and did not comprehensively examine the combined effect of multiple indices [13,78]. Moreover, neither quantification of the specific contribution ratio of specific cooling influencing factors, nor further analyzation of the cooling threshold values of each factor were done. The regression results of the BRT model demonstrate the importance of the cooling impact of spatial factors and clearly describe the quantitative impact difference of the collaborative correlation of multi-dimensional spatial variable systems.

The synergistic cooling effect of blue–green spaces is a comprehensive and complex ecological process. More comprehensive spatial indices related to the spatial correlation index between waterbodies and green spaces must be combined to this study. The actual cooling effect of urban rivers and green spaces is complex, and the blue–green synergistic cooling effect should be examined considering the multi-dimensional spatial factors, including the morphological composition, spatial pattern, and blue–green ecological network elements. In particular, the Cd index represents the connectivity degree of the blue–green ecological landscape and the location of a green space represents the relative position of the green space against rivers. In this study, this index was adopted to comprehensively explain the morphological patterns of green spaces in the waterfront area and to refine the cooling efficiency of the green space types under the framework of the morphological indicators.

Compared with previous studies, this study had formed a set of innovative adopted tools and methods. First, the waterbody and green space data were obtained through artificial visual classification based on a high-precision Google satellite map and modified through artificial surveys. Compared with the software-based computer classification method, this method exhibits a higher precision [79]. In addition, the BRT machine learning method was adopted to quantify the influence of each factor on the LST and the cooling effect characteristics of the rivers on the green space to analyze the relationship between each factor and the LST. Third, to comprehensively study the combined effect of multiple factors, different structural and morphological factors were combined to group a variety of green space types; subsequently, the cooling characteristics of different structural and morphological green space types were discussed through an association analysis with the LST. This study summarized the characteristics of the waterfront green space pattern with the highest cooling effect. This study is in line with the requirements of the control index setting for planning and layout, and can provide optimal suggestions in actual local construction scenarios.

### 4.4. Limitations of the Present Study

Our research was aimed at studying the interaction between the cooling effect of riverfront green spaces and the structural composition of green spaces during a typical summer high-temperature period. The remote sensing data can only provide data in a fixed period of time during the diurnal period. The seasonal variation and differences between diurnal and nocturnal times in the synergistic cooling effect of waterbodies and green spaces has not been analyzed [80,81,82]. Future work can be aimed at performing a comparative analysis of different time variations regarding the cooling effect and obtaining more comprehensive results. Additionally, more diversified methods and tools, such as climate observation data and climate model simulation, should be used to obtain the mechanism of thermo-fluid dynamics for UHI mitigation at different spatial scales.

In addition, only certain widths of rivers have been selected to analyze the cooling effect on the LST of typical blue–green ecological spaces. Moreover, to examine the effect of the built surrounding environmental factors of the waterfront green space on the UCI, only the albedo factor has been considered as the consideration impact index. To conduct a more comprehensive and extensive study, it is necessary to differentiate the composition and structure of various land uses in the study area and to optimize the comprehensive policies to perform adaptive urban development.

## 5. Conclusions

The BRT model was used to estimate the relative contribution ratios of various morphological factors of the waterfront green space in four width-grade river zones to the LST. In all the river-width classifications, the green space coverage (contribution ratio of 32.7–49.2%) and area (contribution ratio of 24.0–29.5%) were the dominant factors affecting the LST. The width of the river and its contribution to the LST increased gradually from Width-I to Width-IV rivers. A larger width of the river corresponded to a greater distance threshold of the synergistic cooling effect. The threshold distance of the blue–green synergistic cooling effect for Width-I to Width-IV rivers was 600 m, 900 m, 1200 m, and 1395 m (the furthest impact distance of the blue–green synergistic cooling effect), respectively.

Through the comparative analysis of the effects of multiple-combination morphological factors of waterfront green spaces, it was noted that a larger green space with higher coverage and higher connectivity, as well as the L-location at the river, corresponded to the least LST. The MG types of green spaces with a high green space coverage and large area generally had low LST distributions. The green spaces located on the leeward side of the river were considerably affected by the river cooling resource and had a higher cooling effect. The green space with the high connectivity degree exhibited enhanced air flow circulation inside the ecological space network, which strengthened the cooling effect. The enhanced connectivity of ecological networks, which can improve urban ventilation, can help optimize the waterfront spatial pattern of green space systems in cities with intensive river networks to mitigate UHIs.

## Figures and Tables

**Table 1:** Multi-dimensional spatial variables to describe the morphology of waterfront green spaces based on the cooling effect.

Impact Variables	Selected Index	Definition and Description
Variables of the spatial morphology of green spaces	Area	Surface area occupied by the green space in units (m^2^).
	Fraction of the vegetation coverage (Fv)	Reflects the vertical coverage of the tree crown; the value of Fv ranges from 0 to 1.
	Landscape shape index (LSI)	Indicates the complexity of shapes, determined by calculating the deviation between the shape of the green space patch and a square of the same area.
	Albedo	Ratio of the surface reflection flux to the incident solar radiation flux on the surface of the green space. Corresponding data obtained through the Landsat 8 data retrieved through the ENVI5.3 software.
Variables of the spatial structure between blue and green spaces	Location of the green space (LG)	Position of green space relative to the river, defined based on the dominant wind direction and position of the green space relative to the river.
	Connectivity degree (Cd)	Connectivity degree of the blue–green ecological network, determined using the dPC index in this study and calculated using the Conefor Sensinode 2.6 software.
	River width (Wd)	Width of each green patch adjacent to the river.
	Distance of the waterfront green space from the riverbank (D)	Distance between the geometric center of the green space and riverbank, representing the influence of the waterbody on the cooling effect of the green space.

**Table 2:** Value interval and meaning of subcategories of green space morphological factors.

Classification Aspects of Variables	Area (ha)					Fv			Cd			LG	
Variable classification	A_1_	A_2_	A_3_	A_4_	A_5_	Fv_1_	Fv_2_	Fv_3_	Cd_1_	Cd_2_	Cd_3_	W	L
Value interval	<1	1–5	5~10	10–20	>20	<0.4	0.4–0.7	0.7–1.0	0–2	2–10	>10		
Meaning	smallest	smaller	intermediate	larger	largest	low	middle	high	low	middle	high	windward	leeward

## Abbreviations

urban heat island	UHI
urban cooling effect	UCI
urban cooling island of green space	UGCI
waterbody cooling island	WCI
land surface temperature	LST
boosted regression trees	BRT
morphological group	MG
marginal effect	ME
normalized difference vegetation index	NDVI
normalized difference built-up index	NDBI
sky view factors	SVF
United States geological survey	USGS
thermal infrared sensor	TIRS
fast line-of-sight atmospheric analysis of spectral hypercubes	FLAASH
radiative transfer equation	RTE
mono-window algorithm	MWA
single-channel method	SCM
ground control points	GCPs
area	A
landscape shape index	LSI
fractional cover values of vegetation space	Fv
width of river	Wd
distance to riverbank	D
probability of connectivity	PC
decrease in the probability of connectivity	dPC
connectivity degree	Cd
location of greenspace	LG
